# Aqua­(2,2′-bipyridine)bis­(4-hydroxy­benzoato)zinc(II)

**DOI:** 10.1107/S1600536809025525

**Published:** 2009-07-08

**Authors:** Bing-Yu Zhang, Jing-Jing Nie, Duan-Jun Xu

**Affiliations:** a Department of Chemistry, Zhejiang University, Hangzhou, 310027, People’s Republic of China

## Abstract

In the title complex, [Zn(C_7_H_5_O_3_)_2_(C_10_H_8_N_2_)(H_2_O)], the Zn^II^ ion is coordinated by two 4-hydroxy­benzoate anions, one 2,2′-bipyridine mol­ecule and one water mol­ecule and displays a distorted octa­hedral geometry. One Zn—O bond [2.5300 (15) Å] is much longer than the others in the mol­ecule. In the crystal structure, the face-to-face separation of 3.547 (9) Å suggests no π–π stacking between parallel bipyridine ring systems, and an extensive O—H⋯O hydrogen-bonding network between the coordinated water molecule, the phenol group and carboxylate O atoms is present.

## Related literature

For general background, see: Xu *et al.* (2007*a* ▶,*b* ▶); Li *et al.* (2005 ▶). For a related structure, see: Kong *et al.* (2008 ▶). For the smaller metal—O—C bond angle  corresponding to the longer coordination bond, see: Li *et al.* (2005 ▶).
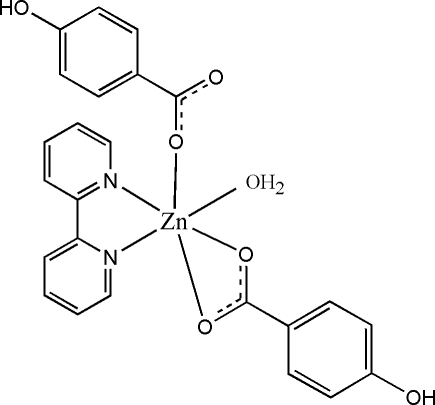


## Experimental

### 

#### Crystal data

[Zn(C_7_H_5_O_3_)_2_(C_10_H_8_N_2_)(H_2_O)]
*M* *_r_* = 513.79
Monoclinic, 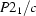

*a* = 10.3549 (12) Å
*b* = 19.524 (3) Å
*c* = 11.5544 (18) Å
β = 107.97 (2)°
*V* = 2221.9 (6) Å^3^
*Z* = 4
Mo *K*α radiation
μ = 1.16 mm^−1^
*T* = 294 K
0.40 × 0.32 × 0.28 mm

#### Data collection

Rigaku R-AXIS RAPID IP diffractometer
Absorption correction: multi-scan (*ABSCOR*; Higashi, 1995 ▶) *T*_min_ = 0.659, *T*_max_ = 0.724
13723 measured reflections
5092 independent reflections
3794 reflections with *I* > 2σ(*I*)
*R*_int_ = 0.028

#### Refinement

*R*[*F* ^2^ > 2σ(*F* ^2^)] = 0.033
*wR*(*F* ^2^) = 0.084
*S* = 1.07
5092 reflections
307 parameters
H-atom parameters constrained
Δρ_max_ = 0.39 e Å^−3^
Δρ_min_ = −0.31 e Å^−3^

### 

Data collection: *PROCESS-AUTO* (Rigaku, 1998 ▶); cell refinement: *PROCESS-AUTO*; data reduction: *CrystalStructure* (Rigaku/MSC, 2002 ▶); program(s) used to solve structure: *SIR92* (Altomare *et al.*, 1993 ▶); program(s) used to refine structure: *SHELXL97* (Sheldrick, 2008 ▶); molecular graphics: *ORTEP-3* (Farrugia, 1997 ▶); software used to prepare material for publication: *WinGX* (Farrugia, 1999 ▶).

## Supplementary Material

Crystal structure: contains datablocks I, global. DOI: 10.1107/S1600536809025525/hk2727sup1.cif

Structure factors: contains datablocks I. DOI: 10.1107/S1600536809025525/hk2727Isup2.hkl

Additional supplementary materials:  crystallographic information; 3D view; checkCIF report

## Figures and Tables

**Table 1:** Selected bond lengths (Å)

Zn—O1	2.5300 (15)
Zn—O2	2.0045 (14)
Zn—O4	2.0607 (14)
Zn—O7	2.1375 (15)
Zn—N1	2.0986 (18)
Zn—N2	2.1275 (17)

**Table 2:** Hydrogen-bond geometry (Å, °)

*D*—H⋯*A*	*D*—H	H⋯*A*	*D*⋯*A*	*D*—H⋯*A*
O3—H3*A*⋯O1^i^	0.94	1.64	2.565 (2)	169
O6—H6*A*⋯O4^ii^	0.87	1.81	2.668 (2)	168
O7—H7*A*⋯O3^iii^	0.90	1.93	2.813 (2)	168
O7—H7*B*⋯O5	0.95	1.73	2.636 (2)	158

Symmetry codes: (i) 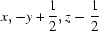; (ii) 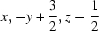; (iii) 
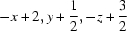
.

**Fig. 1.**
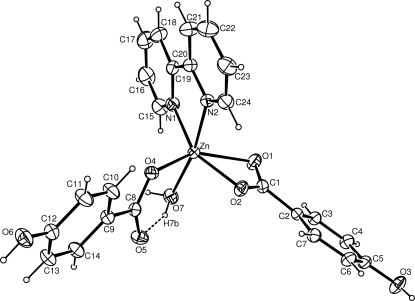
The molecular structure of the title complex with 30% probability displacement ellipsoids (arbitrary spheres for H atoms). The dashed line indicates the intramolecular hydrogen bonding.

## supplementary crystallographic information

### Comment

As a part of our ongoing investigation on the nature of π-π stacking (Xu *et al.*, 2007*a*, 2007*b*; Li *et al.*, 2005), the title complex was prepared in the laboratory and its crystal structure is reported herein.

The molecular structure of the title complex is shown in Fig. 1. The Zn^II^ cation is coordinated by two 4-hydroxybenzoate anions, one 2,2-bipyridine and one water molecules with a distorted octahedral geometry. The Zn—O1 [2.5300 (15) Å] bond is much longer than the other Zn—O bonds in the molecule (Table 1), but the smaller Zn–O2–C1 [103.38 (13)°] bond angle suggests the existence of a genuine bonding between O1 and Zn atoms (Li *et al.* 2005). This is similar to that in aqua-(4-hydroxybenzoato)(4-hydroxybenzoato) (1,10-phenanthroline)zinc monohydrate (Kong *et al.*, 2008).

In the crystal structure, extensive O—H···O hydrogen bonding occurs (Table 2) and the longer face-to-face separation of 3.547 (9) Å between parallel bipyridine ligands, related by an inversion center, suggests no π-π stacking.

### Experimental

ZnCl_2_ (0.136 g, 1 mmol), 4-hydroxybenzoic acid (0.14 g, 1 mmol), Na_2_CO_3_ (0.053 g, 0.5 mmol) and 2,2-bipyridine (0.156 g, 1 mmol) were dissolved in a water/ethanol solution (20 ml, 2:3). The mixture was refluxed for 4 h, and then cooled to room temperature and filtered. Colorless single crystals were obtained from the filtrate after 10 d.

### Refinement

H atoms of hydroxy groups and water molecules were located in a difference Fourier map and refined as riding in their as-found relative positions, *U*_iso_(H) = 1.5*U*_eq_(O). Other H atoms were placed in calculated positions with C—H = 0.93 Å, and refined in riding mode with *U*_iso_(H) = 1.2*U*_eq_(C).

### Crystal data

**Table:**

[Zn(C_7_H_5_O_3_)_2_(C_10_H_8_N_2_)(H_2_O)]	*F*(000) = 1056
*M**_r_* = 513.79	*D*_x_ = 1.536 Mg m^−^^3^
Monoclinic, *P*2_1_/*c*	Mo *K*α radiation, λ = 0.71073 Å
Hall symbol: -P 2ybc	Cell parameters from 8786 reflections
*a* = 10.3549 (12) Å	θ = 2.8–25.0°
*b* = 19.524 (3) Å	µ = 1.16 mm^−^^1^
*c* = 11.5544 (18) Å	*T* = 294 K
β = 107.97 (2)°	Prism, colorless
*V* = 2221.9 (6) Å^3^	0.40 × 0.32 × 0.28 mm
*Z* = 4	

### Data collection

**Table:**

Rigaku R-AXIS RAPID IP diffractometer	5092 independent reflections
Radiation source: fine-focus sealed tube	3794 reflections with *I* > 2σ(*I*)
graphite	*R*_int_ = 0.028
Detector resolution: 10.00 pixels mm^-1^	θ_max_ = 27.5°, θ_min_ = 2.1°
ω scans	*h* = −13→12
Absorption correction: multi-scan (*ABSCOR*; Higashi, 1995)	*k* = −16→25
*T*_min_ = 0.659, *T*_max_ = 0.724	*l* = −15→14
13723 measured reflections	

### Refinement

**Table:**

Refinement on *F*^2^	Primary atom site location: structure-invariant direct methods
Least-squares matrix: full	Secondary atom site location: difference Fourier map
*R*[*F*^2^ > 2σ(*F*^2^)] = 0.033	Hydrogen site location: inferred from neighbouring sites
*wR*(*F*^2^) = 0.084	H-atom parameters constrained
*S* = 1.07	*w* = 1/[σ^2^(*F*_o_^2^) + (0.0381*P*)^2^ + 0.2291*P*] where *P* = (*F*_o_^2^ + 2*F*_c_^2^)/3
5092 reflections	(Δ/σ)_max_ = 0.001
307 parameters	Δρ_max_ = 0.39 e Å^−^^3^
0 restraints	Δρ_min_ = −0.30 e Å^−^^3^

### Special details

**Table:**

Geometry. All e.s.d.'s (except the e.s.d. in the dihedral angle between two l.s. planes) are estimated using the full covariance matrix. The cell e.s.d.'s are taken into account individually in the estimation of e.s.d.'s in distances, angles and torsion angles; correlations between e.s.d.'s in cell parameters are only used when they are defined by crystal symmetry. An approximate (isotropic) treatment of cell e.s.d.'s is used for estimating e.s.d.'s involving l.s. planes.
Refinement. Refinement of *F*^2^ against ALL reflections. The weighted *R*-factor *wR* and goodness of fit *S* are based on *F*^2^, conventional *R*-factors *R* are based on *F*, with *F* set to zero for negative *F*^2^. The threshold expression of *F*^2^ > σ(*F*^2^) is used only for calculating *R*-factors(gt) *etc*. and is not relevant to the choice of reflections for refinement. *R*-factors based on *F*^2^ are statistically about twice as large as those based on *F*, and *R*- factors based on ALL data will be even larger.

### Fractional atomic coordinates and isotropic or equivalent isotropic displacement parameters (Å^2^)

**Table:**

	*x*	*y*	*z*	*U*_iso_*/*U*_eq_	
Zn	0.72142 (2)	0.523813 (12)	0.76441 (2)	0.03203 (9)	
N1	0.71413 (18)	0.55972 (9)	0.93348 (15)	0.0364 (4)	
N2	0.50939 (18)	0.52685 (8)	0.74053 (16)	0.0326 (4)	
O1	0.79866 (17)	0.40502 (8)	0.84339 (14)	0.0472 (4)	
O2	0.72447 (15)	0.44341 (7)	0.65740 (13)	0.0390 (4)	
O3	0.90448 (17)	0.14372 (8)	0.55801 (14)	0.0503 (4)	
H3A	0.8612	0.1314	0.4770	0.075*	
O4	0.69289 (14)	0.60029 (7)	0.63604 (13)	0.0349 (3)	
O5	0.88734 (15)	0.59867 (8)	0.59074 (13)	0.0419 (4)	
O6	0.53361 (16)	0.81021 (8)	0.20163 (14)	0.0473 (4)	
H6A	0.5940	0.8348	0.1816	0.071*	
O7	0.93531 (15)	0.53817 (7)	0.80380 (14)	0.0418 (4)	
H7A	0.9755	0.5746	0.8475	0.063*	
H7B	0.9281	0.5502	0.7227	0.063*	
C1	0.7783 (2)	0.39661 (10)	0.73229 (19)	0.0329 (5)	
C2	0.8162 (2)	0.33111 (10)	0.68419 (18)	0.0293 (4)	
C3	0.8999 (2)	0.28391 (10)	0.76110 (18)	0.0329 (5)	
H3	0.9367	0.2942	0.8434	0.039*	
C4	0.9297 (2)	0.22205 (11)	0.71799 (18)	0.0359 (5)	
H4	0.9871	0.1911	0.7706	0.043*	
C5	0.8738 (2)	0.20608 (10)	0.59584 (19)	0.0342 (5)	
C6	0.7894 (2)	0.25250 (11)	0.51718 (19)	0.0359 (5)	
H6	0.7508	0.2417	0.4354	0.043*	
C7	0.7633 (2)	0.31490 (10)	0.56143 (18)	0.0329 (5)	
H7	0.7093	0.3467	0.5081	0.040*	
C8	0.7700 (2)	0.61981 (10)	0.57269 (18)	0.0315 (5)	
C9	0.7101 (2)	0.67051 (10)	0.47486 (18)	0.0323 (5)	
C10	0.5709 (2)	0.67839 (12)	0.4277 (2)	0.0418 (5)	
H10	0.5143	0.6516	0.4579	0.050*	
C11	0.5143 (2)	0.72511 (12)	0.3370 (2)	0.0442 (6)	
H11	0.4205	0.7292	0.3059	0.053*	
C12	0.5968 (2)	0.76610 (11)	0.29181 (19)	0.0360 (5)	
C13	0.7366 (2)	0.75963 (11)	0.3390 (2)	0.0402 (5)	
H13	0.7928	0.7875	0.3103	0.048*	
C14	0.7924 (2)	0.71194 (11)	0.42857 (19)	0.0386 (5)	
H14	0.8863	0.7073	0.4585	0.046*	
C15	0.8215 (3)	0.56939 (13)	1.0329 (2)	0.0484 (6)	
H15	0.9075	0.5584	1.0290	0.058*	
C16	0.8100 (3)	0.59469 (14)	1.1398 (2)	0.0607 (7)	
H16	0.8866	0.6009	1.2069	0.073*	
C17	0.6831 (3)	0.61079 (14)	1.1463 (2)	0.0614 (8)	
H17	0.6727	0.6286	1.2176	0.074*	
C18	0.5722 (3)	0.60022 (13)	1.0462 (2)	0.0532 (7)	
H18	0.4855	0.6105	1.0492	0.064*	
C19	0.5899 (2)	0.57419 (10)	0.9407 (2)	0.0367 (5)	
C20	0.4749 (2)	0.55895 (11)	0.82961 (19)	0.0359 (5)	
C21	0.3423 (3)	0.57615 (13)	0.8165 (2)	0.0528 (6)	
H21	0.3199	0.5984	0.8788	0.063*	
C22	0.2426 (3)	0.55975 (15)	0.7089 (3)	0.0590 (7)	
H22	0.1526	0.5715	0.6981	0.071*	
C23	0.2768 (3)	0.52628 (13)	0.6190 (2)	0.0512 (6)	
H23	0.2111	0.5142	0.5468	0.061*	
C24	0.4112 (2)	0.51086 (12)	0.6382 (2)	0.0424 (5)	
H24	0.4351	0.4882	0.5770	0.051*	

### Atomic displacement parameters (Å^2^)

**Table:**

	*U*^11^	*U*^22^	*U*^33^	*U*^12^	*U*^13^	*U*^23^
Zn	0.03499 (15)	0.02836 (14)	0.03540 (15)	0.00098 (10)	0.01475 (11)	−0.00214 (10)
N1	0.0381 (11)	0.0359 (10)	0.0350 (10)	−0.0019 (8)	0.0111 (8)	−0.0014 (8)
N2	0.0357 (10)	0.0325 (10)	0.0333 (9)	−0.0021 (7)	0.0159 (8)	−0.0029 (8)
O1	0.0717 (12)	0.0333 (9)	0.0397 (9)	0.0048 (8)	0.0215 (8)	−0.0022 (7)
O2	0.0454 (9)	0.0282 (8)	0.0428 (9)	0.0082 (7)	0.0128 (7)	0.0003 (7)
O3	0.0730 (12)	0.0339 (9)	0.0415 (9)	0.0193 (8)	0.0141 (8)	−0.0036 (7)
O4	0.0375 (8)	0.0302 (8)	0.0424 (8)	0.0048 (6)	0.0204 (7)	0.0063 (6)
O5	0.0306 (9)	0.0527 (10)	0.0440 (9)	0.0025 (7)	0.0140 (7)	0.0054 (7)
O6	0.0439 (10)	0.0430 (9)	0.0572 (10)	−0.0002 (7)	0.0186 (8)	0.0184 (8)
O7	0.0359 (9)	0.0431 (9)	0.0450 (9)	−0.0054 (7)	0.0103 (7)	0.0000 (7)
C1	0.0329 (11)	0.0263 (11)	0.0414 (12)	−0.0033 (9)	0.0142 (10)	−0.0021 (9)
C2	0.0296 (11)	0.0252 (10)	0.0364 (11)	−0.0009 (8)	0.0149 (9)	0.0000 (8)
C3	0.0352 (12)	0.0324 (11)	0.0311 (11)	0.0012 (9)	0.0102 (9)	−0.0009 (9)
C4	0.0408 (13)	0.0321 (12)	0.0341 (11)	0.0101 (9)	0.0105 (9)	0.0062 (9)
C5	0.0407 (13)	0.0275 (11)	0.0387 (12)	0.0044 (9)	0.0187 (10)	−0.0004 (9)
C6	0.0410 (13)	0.0375 (12)	0.0293 (11)	0.0051 (9)	0.0109 (9)	−0.0023 (9)
C7	0.0339 (12)	0.0301 (11)	0.0357 (12)	0.0064 (9)	0.0121 (9)	0.0054 (9)
C8	0.0338 (12)	0.0277 (11)	0.0337 (11)	−0.0056 (9)	0.0115 (9)	−0.0065 (9)
C9	0.0339 (12)	0.0300 (11)	0.0345 (11)	−0.0029 (9)	0.0126 (9)	0.0000 (9)
C10	0.0355 (13)	0.0440 (13)	0.0496 (14)	−0.0061 (10)	0.0186 (11)	0.0098 (11)
C11	0.0295 (12)	0.0501 (14)	0.0534 (14)	−0.0022 (10)	0.0133 (10)	0.0146 (11)
C12	0.0407 (13)	0.0311 (11)	0.0381 (12)	−0.0024 (9)	0.0151 (10)	0.0023 (9)
C13	0.0374 (13)	0.0403 (13)	0.0460 (14)	−0.0113 (10)	0.0172 (10)	0.0052 (10)
C14	0.0305 (12)	0.0433 (13)	0.0426 (13)	−0.0080 (9)	0.0121 (10)	0.0016 (10)
C15	0.0475 (15)	0.0535 (16)	0.0415 (14)	−0.0066 (11)	0.0099 (11)	0.0007 (12)
C16	0.069 (2)	0.0658 (19)	0.0397 (15)	−0.0206 (15)	0.0060 (13)	−0.0063 (13)
C17	0.087 (2)	0.0605 (18)	0.0409 (15)	−0.0099 (15)	0.0252 (15)	−0.0159 (13)
C18	0.0646 (18)	0.0538 (16)	0.0480 (15)	0.0030 (13)	0.0274 (13)	−0.0110 (12)
C19	0.0453 (13)	0.0275 (11)	0.0409 (12)	0.0003 (9)	0.0186 (10)	−0.0009 (9)
C20	0.0392 (13)	0.0331 (12)	0.0399 (12)	0.0009 (9)	0.0187 (10)	0.0001 (10)
C21	0.0457 (15)	0.0651 (17)	0.0541 (16)	0.0074 (12)	0.0249 (13)	−0.0058 (13)
C22	0.0365 (14)	0.075 (2)	0.0681 (19)	0.0054 (13)	0.0194 (13)	0.0037 (15)
C23	0.0399 (14)	0.0640 (18)	0.0464 (14)	−0.0067 (12)	0.0082 (11)	0.0046 (13)
C24	0.0448 (14)	0.0463 (14)	0.0377 (12)	−0.0075 (11)	0.0151 (11)	−0.0042 (10)

### Geometric parameters (Å, °)

**Table:**

Zn—O1	2.5300 (15)	C7—H7	0.9300
Zn—O2	2.0045 (14)	C8—C9	1.486 (3)
Zn—O4	2.0607 (14)	C9—C10	1.383 (3)
Zn—O7	2.1375 (15)	C9—C14	1.396 (3)
Zn—N1	2.0986 (18)	C10—C11	1.377 (3)
Zn—N2	2.1275 (17)	C10—H10	0.9300
N1—C15	1.343 (3)	C11—C12	1.384 (3)
N1—C19	1.344 (3)	C11—H11	0.9300
N2—C24	1.337 (3)	C12—C13	1.386 (3)
N2—C20	1.344 (3)	C13—C14	1.379 (3)
O1—C1	1.246 (2)	C13—H13	0.9300
O2—C1	1.264 (2)	C14—H14	0.9300
O3—C5	1.364 (2)	C15—C16	1.369 (3)
O3—H3A	0.9362	C15—H15	0.9300
O4—C8	1.295 (2)	C16—C17	1.375 (4)
O5—C8	1.239 (2)	C16—H16	0.9300
O6—C12	1.355 (2)	C17—C18	1.371 (4)
O6—H6A	0.8744	C17—H17	0.9300
O7—H7A	0.8971	C18—C19	1.384 (3)
O7—H7B	0.9465	C18—H18	0.9300
C1—C2	1.494 (3)	C19—C20	1.487 (3)
C2—C3	1.384 (3)	C20—C21	1.376 (3)
C2—C7	1.390 (3)	C21—C22	1.386 (4)
C3—C4	1.377 (3)	C21—H21	0.9300
C3—H3	0.9300	C22—C23	1.364 (4)
C4—C5	1.386 (3)	C22—H22	0.9300
C4—H4	0.9300	C23—C24	1.373 (3)
C5—C6	1.386 (3)	C23—H23	0.9300
C6—C7	1.380 (3)	C24—H24	0.9300
C6—H6	0.9300		
			
O1—Zn—O2	56.03 (5)	O5—C8—C9	120.47 (18)
O1—Zn—O4	152.01 (5)	O4—C8—C9	116.13 (18)
O1—Zn—O7	81.49 (6)	C10—C9—C14	118.02 (19)
O1—Zn—N1	93.94 (6)	C10—C9—C8	120.87 (18)
O1—Zn—N2	105.73 (6)	C14—C9—C8	121.11 (19)
O2—Zn—O4	98.60 (6)	C11—C10—C9	121.4 (2)
O2—Zn—O7	91.23 (6)	C11—C10—H10	119.3
O2—Zn—N1	147.97 (7)	C9—C10—H10	119.3
O2—Zn—N2	98.85 (6)	C10—C11—C12	120.2 (2)
O4—Zn—O7	88.12 (6)	C10—C11—H11	119.9
O4—Zn—N1	112.93 (6)	C12—C11—H11	119.9
O4—Zn—N2	88.48 (6)	O6—C12—C11	116.7 (2)
N1—Zn—O7	95.19 (7)	O6—C12—C13	123.98 (19)
N1—Zn—N2	77.25 (7)	C11—C12—C13	119.3 (2)
N2—Zn—O7	169.74 (6)	C14—C13—C12	120.1 (2)
Zn—O1—C1	79.34 (12)	C14—C13—H13	119.9
Zn—O2—C1	103.38 (13)	C12—C13—H13	119.9
Zn—O4—C8	130.04 (13)	C13—C14—C9	121.0 (2)
C15—N1—C19	118.2 (2)	C13—C14—H14	119.5
C15—N1—Zn	125.83 (16)	C9—C14—H14	119.5
C19—N1—Zn	115.97 (14)	N1—C15—C16	122.9 (2)
C24—N2—C20	118.50 (19)	N1—C15—H15	118.6
C24—N2—Zn	125.52 (15)	C16—C15—H15	118.6
C20—N2—Zn	114.88 (14)	C15—C16—C17	118.8 (2)
C5—O3—H3A	117.6	C15—C16—H16	120.6
C12—O6—H6A	109.7	C17—C16—H16	120.6
Zn—O7—H7A	119.6	C18—C17—C16	119.0 (2)
Zn—O7—H7B	93.5	C18—C17—H17	120.5
H7A—O7—H7B	104.0	C16—C17—H17	120.5
O1—C1—O2	120.60 (19)	C17—C18—C19	119.6 (2)
O1—C1—C2	121.04 (19)	C17—C18—H18	120.2
O2—C1—C2	118.36 (18)	C19—C18—H18	120.2
C3—C2—C7	118.38 (18)	N1—C19—C18	121.4 (2)
C3—C2—C1	121.02 (18)	N1—C19—C20	115.53 (19)
C7—C2—C1	120.54 (18)	C18—C19—C20	123.0 (2)
C4—C3—C2	121.16 (19)	N2—C20—C21	121.4 (2)
C4—C3—H3	119.4	N2—C20—C19	115.06 (19)
C2—C3—H3	119.4	C21—C20—C19	123.5 (2)
C3—C4—C5	119.73 (19)	C20—C21—C22	119.0 (2)
C3—C4—H4	120.1	C20—C21—H21	120.5
C5—C4—H4	120.1	C22—C21—H21	120.5
O3—C5—C4	117.50 (18)	C23—C22—C21	119.8 (2)
O3—C5—C6	122.40 (19)	C23—C22—H22	120.1
C4—C5—C6	120.10 (19)	C21—C22—H22	120.1
C7—C6—C5	119.36 (19)	C22—C23—C24	118.1 (2)
C7—C6—H6	120.3	C22—C23—H23	120.9
C5—C6—H6	120.3	C24—C23—H23	120.9
C6—C7—C2	121.23 (19)	N2—C24—C23	123.2 (2)
C6—C7—H7	119.4	N2—C24—H24	118.4
C2—C7—H7	119.4	C23—C24—H24	118.4
O5—C8—O4	123.39 (19)		

### Hydrogen-bond geometry (Å, °)

**Table:**

*D*—H···*A*	*D*—H	H···*A*	*D*···*A*	*D*—H···*A*
O3—H3A···O1^i^	0.94	1.64	2.565 (2)	169
O6—H6A···O4^ii^	0.87	1.81	2.668 (2)	168
O7—H7A···O3^iii^	0.90	1.93	2.813 (2)	168
O7—H7B···O5	0.95	1.73	2.636 (2)	158

Symmetry codes: (i) *x*, −*y*+1/2, *z*−1/2; (ii) *x*, −*y*+3/2, *z*−1/2; (iii) −*x*+2, *y*+1/2, −*z*+3/2.
